# Intergenerational Relationships, Social Support, and Psychological Well-Being Among Korean Older Adults

**DOI:** 10.1093/geroni/igab046.1172

**Published:** 2021-12-17

**Authors:** Yooumi Lee, Janet Wilmoth

**Affiliations:** Syracuse University, Syracuse, New York, United States

## Abstract

Using longitudinal data from the 2006 to 2018 Korean Longitudinal Study of Aging, this study explores depression trajectories among individuals who are 60 or older with at least one living adult child at baseline. We estimated linear growth curve models of depression trajectories separately for married, unmarried and widowed using the Center for Epidemiologic Studies Depression Scale (CES-D). Results indicate that declining health and recent widowhood are positively related to depressive symptoms. Satisfactory intergenerational relationships and social support in the form of caregiving decrease depressive symptoms of older parents, especially among the widowed. Having at least one son and a first-born daughter positively impact psychological well-being of older parents. A son was particularly important for those who are widowed. We conclude that the psychological benefits of intergenerational relationships and social support are contingent upon the vulnerability of Korean older adults and discuss the implications for public policy.

